# A Web-Based Program Improves Physical Activity Outcomes in a Primary Care Angina Population: Randomized Controlled Trial

**DOI:** 10.2196/jmir.3340

**Published:** 2014-09-12

**Authors:** Reena Devi, John Powell, Sally Singh

**Affiliations:** ^1^Coventry UniversityApplied Research Centre in Health and Lifestyle InterventionsCoventryUnited Kingdom; ^2^University of OxfordNuffield Department of Primary Care Health SciencesOxfordUnited Kingdom; ^3^Collaboration for Leadership in Applied Health Research and Care East Midlands (CLAHRC EM)University Hospitals of Leicester, Glenfield HospitalCentre for Exercise and Rehabilitation ScienceLeicesterUnited Kingdom

**Keywords:** stable angina, cardiac rehabilitation, Web-based interventions, secondary prevention, primary health care, physical activity

## Abstract

**Background:**

Angina affects more than 50 million people worldwide. Secondary prevention interventions such as cardiac rehabilitation are not widely available for this population. An Internet-based version could offer a feasible alternative.

**Objective:**

Our aim was to examine the effectiveness of a Web-based cardiac rehabilitation program for those with angina.

**Methods:**

We conducted a randomized controlled trial, recruiting those diagnosed with angina from general practitioners (GPs) in primary care to an intervention or control group. Intervention group participants were offered a 6-week Web-based rehabilitation program (“ActivateYourHeart”). The program was introduced during a face-to-face appointment and then delivered via the Internet (no further face-to-face contact). The program contained information about the secondary prevention of coronary heart disease (CHD) and set each user goals around physical activity, diet, managing emotions, and smoking. Performance against goals was reviewed throughout the program and goals were then reset/modified. Participants completed an online exercise diary and communicated with rehabilitation specialists through an email link/synchronized chat room. Participants in the control group continued with GP treatment as usual, which consisted of being placed on a CHD register and attending an annual review. Outcomes were measured at 6-week and 6-month follow-ups during face-to-face assessments. The primary outcome measure was change in daily steps at 6 weeks, measured using an accelerometer. Secondary outcome measures were energy expenditure (EE), duration of sedentary activity (DSA), duration of moderate activity (DMA), weight, diastolic/systolic blood pressure, and body fat percentage. Self-assessed questionnaire outcomes included fat/fiber intake, anxiety/depression, self-efficacy, and quality of life (QOL).

**Results:**

A total of 94 participants were recruited and randomized to the intervention (n=48) or the usual care (n=46) group; 84 and 73 participants completed the 6-week and 6-month follow-ups, respectively. The mean number of log-ins to the program was 18.68 (SD 13.13, range 1-51), an average of 3 log-ins per week per participant. Change in daily steps walked at the 6-week follow-up was +497 (SD 2171) in the intervention group and –861 (SD 2534) in the control group (95% CI 263-2451, *P*=.02). Significant intervention effects were observed at the 6-week follow-up in EE (+43.94 kcal, 95% CI 43.93-309.98, *P*=.01), DSA (–7.79 minutes, 95% CI –55.01 to –7.01, *P*=.01), DMA (+6.31 minutes, 95% CI 6.01-51.20, *P*=.01), weight (–0.56 kg, 95% CI –1.78 to –0.15, *P*=.02), self-efficacy (95% CI 0.30-4.79, *P*=.03), emotional QOL score (95% CI 0.01-0.54, *P*=.04), and angina frequency (95% CI 8.57-35.05, *P*=.002). Significant benefits in angina frequency (95% CI 1.89-29.41, *P*=.02) and social QOL score (95% CI 0.05-0.54, *P*=.02) were also observed at the 6-month follow-up.

**Conclusions:**

An Internet-based secondary prevention intervention could be offered to those with angina. A larger pragmatic trial is required to provide definitive evidence of effectiveness and cost-effectiveness.

**Trial Registration:**

International Standard Randomized Controlled Trial Number (ISRCTN): 90110503; http://www.controlled-trials.com/ISRCTN90110503/ISRCTN90110503 (Archived by WebCite at http://www.webcitation.org/6RYVOQFKM).

## Introduction

The impact of angina is significant to both the individual [1] and to the health service [2]. Cardiac rehabilitation is recommended for individuals with angina in many international guidelines [3], but capacity to accommodate these individuals is limited and those with a recent cardiac event take priority. Recent data suggest that angina patients constitute only 4% of the referrals to rehabilitation, and almost 20% of programs do not accept those with angina [4].

There is a broad spectrum of interventions that may constitute a rehabilitation program, from fully supervised sessions to more remote home-based services. It is largely these self-directed home-based programs that have been tested in the angina population. A meta-analysis of 7 trials [5] demonstrated that psychoeducational interventions delivered via a trained professional significantly reduced medication use, physical limitations, and disease perception in angina populations. In addition, the angina population have been considered previously with a manual-based approach, The Angina Plan [6,7]; however, this has not been widely adopted [4]. A small number of trials have studied the effectiveness of secondary prevention interventions for coronary heart disease (CHD) delivered via the Internet. A recent Canadian study [8] evaluated a 6-month Web-based physical activity program for patients who had undergone percutaneous coronary revascularization. The study did not report baseline scores, but the authors reported higher levels of physical activity in the intervention group compared to the control group. The change in physical activity was reported from the 6- to 12-month follow-ups in the intervention group and this was significant compared to the control group. Recently a study conducted in Norway assessed the effectiveness of an Internet- and mobile phone-based intervention for physical activity as an extension of face-to-face cardiac rehabilitation [9]. The study demonstrated significantly higher physical activity levels in the intervention group compared to a control group at 3-month follow-up. However, the study is somewhat limited by the small sample size at follow-up (n=7) and the self-reported measure of physical activity. The value of Web-based interventions and physical activity promotion has also been investigated by Van den Berg et al in a systematic review [10]. Van den Berg et al reviewed 10 articles and reported online interventions are effective in improving physical activity levels [10]. This review emphasized the need to measure physical activity using objective measures. Positive findings have been reported from research measuring physical activity objectively when evaluating Web-based physical activity interventions [11,12].

We developed an interactive password-protected website specifically for individuals with CHD, which is a comprehensive educational package that aims to improve health behaviors related to CHD. The Internet allows for the delivery of a standard intervention that is not geographically or time restrained. It is intended that this intervention could be offered to those not routinely included within traditional cardiac rehabilitation, such as those with stable angina. The purpose of this study was to assess the clinical effectiveness of this independent Internet-delivered self-managed “rehabilitation” program in a population with chronic stable angina in a primary care setting. Because we were studying the efficacy of a novel intervention for which we had only limited previous data, our primary hypothesis was nondirectional and was “users of a Web-based cardiac rehabilitation program would alter their coronary risk factors compared to those receiving treatment as usual (control group).”

## Methods

### Study Design and Randomization

A randomized controlled trial with 2 parallel group arms was conducted (ISRCTN 90110503). The 2 groups consisted of the intervention group and the treatment-as-usual control group. A computerized block randomization list was produced by our departmental statistician. Allocation concealment was achieved by sequentially numbered sealed envelopes, opened after baseline data collection for each participant by the researcher carrying out the fieldwork (RD). Participants and the outcome assessor were not blinded to group allocation.

### Recruitment and Participants

Participants were recruited offline from 9 primary care general practitioners (GPs) in 1 region of England. Participants were selected from CHD registers by a GP or practice nurse. Individuals were invited to participate if they had a confirmed diagnosis of stable angina, were able to read and speak fluent English, had regular access to the Internet, were computer literate, and had not had conventional cardiac rehabilitation within the previous year. Individuals were excluded if they had unstable angina, significant cardiac arrhythmia, any comorbidities preventing physical activity, or were severely anxious/ depressed. Severely anxious/depressed patients were excluded by eliminating anyone with a history of being prescribed medication for either anxiety or depression. Participants were not banned from attending conventional rehabilitation; however, if a participant was offered rehabilitation or any other secondary prevention intervention during the course of the study, they were excluded because this was considered a breach of study design. At each study follow-up, this was asked and recorded in the study notes. Participant recruitment and outcome follow-ups were carried out from September 2008 to February 2010.

### Outcome Measures and Data Collection

Both primary and secondary outcome measures were collected at baseline, 6 weeks after randomization, and then 6 months after the 6-week follow-up by a researcher (RD) visiting the participants at home. Participant follow-up continued until October 2010.

### Primary Outcome Measure

The primary outcome measure was daily average step count change at 6-week follow-up. This was measured using Sensewear Pro 3 accelerometer technology, a nondisplay multisensor monitor. This monitor uses physiological signals, bodily movement, and in-built algorithms to estimate physical activity. Participants wore the monitor on the right upper arm for 2 weekdays (12 hours per day) at baseline and at the 6-week and 6-month follow-ups. Reliability and accuracy of this technology has been established in healthy individuals [13] and unhealthy individuals [14,15].

### Secondary Outcome Measures

Secondary outcome measures included energy expenditure (EE), duration of sedentary activity (DSA), and duration of moderate activity (DMA); these were measured using the same accelerometer that measured the primary outcome. Participants wore the accelerometer on the right upper arm for 2 weekdays (12 hours per day) at baseline and at the 6-week and 6-month follow-ups. Weight, diastolic (DBP) and systolic blood pressure (SBP), and body fat percentage were measured using conventional instruments. Other outcomes were fat and fiber intake, anxiety and depression, self-efficacy, and health-related quality of life (QOL). Fat and fiber intake was measured using the Dietary Instrument for Nutritional Evaluation [16], which is a validated measure to assess fat and fiber intake [16]. This measure contained 19 groups of foods representing fat and fiber in a typical UK diet, and involved participants choosing the frequency of food groups consumed from multiple-choice answers. Anxiety and depression was assessed using the Hospital Anxiety and Depression Scale (HADS) [17], which is a 14-item validated measure of anxiety and depression [18]. This measure is also a reliable and valid instrument for use in a stable coronary population [19]. Self-efficacy was measured using The General Self-Efficacy Scale, a reliable and valid measure of self-efficacy [20], which is comprised of 10 items scored on a 4-point scale. The developers acknowledge it is a general scale and, therefore, suggest additional specific items can be added [21]. In this study, self-efficacy of exercise (3 items), knowledge of heart disease (1 item), and eating a healthy diet (1 item) were added as extra items to the scale. The final score of all items was used to describe the overall self-efficacy of participants; higher scores reflected greater self-efficacy. Health-related QOL was assessed using The MacNew questionnaire [22] and The Seattle Angina Questionnaire (SAQ) [23], of which both are CHD specific. The MacNew questionnaire consists of 27 items measuring perceived quality of emotional, physical, and social health. Each item was scored on a 7-point scale with lower scores corresponding to impaired QOL. This has been reported to be a valid and reliable measure, sensitive to changes in health-related QOL [22], and reliable/valid for use in angina patients [24]. The SAQ questionnaire comprises 19 questions that constitute 5 subscales: physical limitations, angina stability, angina frequency, treatment satisfaction, and disease perception. Lower scores indicate poorer health status and higher scores indicate better health. This measure has undergone validity and reliability testing [23]. In the intervention group, we also monitored the number of log-ins to the online program. This information was available from the administration side of the intervention.

### Procedure

Eligible individuals were sent a postal invitation and those who replied with an interest in participating were contacted. Prospective participants were telephoned by the study researcher to check trial suitability and to arrange an initial home visit. The initial home visit was arranged at a time most convenient for the participant. During the home visit, the researcher (RD) explained that the purpose of the study was to investigate the effectiveness of a Web-based intervention, described study details, took participant consent, and carried out the physical baseline outcome measures (weight, blood pressure, and body fat percentage). Because the initial home visit was arranged at a time most convenient for the participant, it was not possible to control for factors such as time of day, whether the participant felt rested, nor whether the participant was alone or not during the time of measurement. During this initial meeting, participants were also given an accelerometer and a questionnaire pack. Each participant was instructed to wear the monitor for 2 weekdays (12 hours per day) and to complete the questionnaires (paper-based questionnaires). This initial meeting lasted approximately 40 minutes. After all baseline measures were collected, the researcher (RD) randomized each participant, telling each participant which group they had been allocated to. Those in the Web-based cardiac rehabilitation group received a face-to-face introductory session from the researcher (RD). This involved registering the individual, creating a unique username/password, and demonstrating how to use the program. Intervention group participants were told to log in to the program daily to record their daily physical activity. The control group did not receive any intervention and continued care as usual. Study outcome measures were repeated at the 6-week and 6-month follow-ups. Participants were not paid to take part in this trial.

### Intervention

The intervention was delivered via the Internet and called “ActivateYourHeart” [25], a secure and password-protected site designed for participants to use at home. The program was developed at the University Hospitals of Leicester NHS Trust and coproduced with health care professionals, a software development team (HARK2), and a group of patients/members of the public. Development of the site was an interactive and iterative process, involving patients providing input and feedback on different versions of the website, including feedback on website content, layout, visual features, and ease of website navigation. The program aimed to improve patients’ cardiac risk profile within 4 stages and was designed to be completed within 6 weeks. The intervention used the following behavior change techniques [26]: setting/reviewing behavioral goals, self-monitoring, feedback on behavior, graded tasks, social reward, providing information about health consequences, and reducing negative emotions.

**Figure 1:**
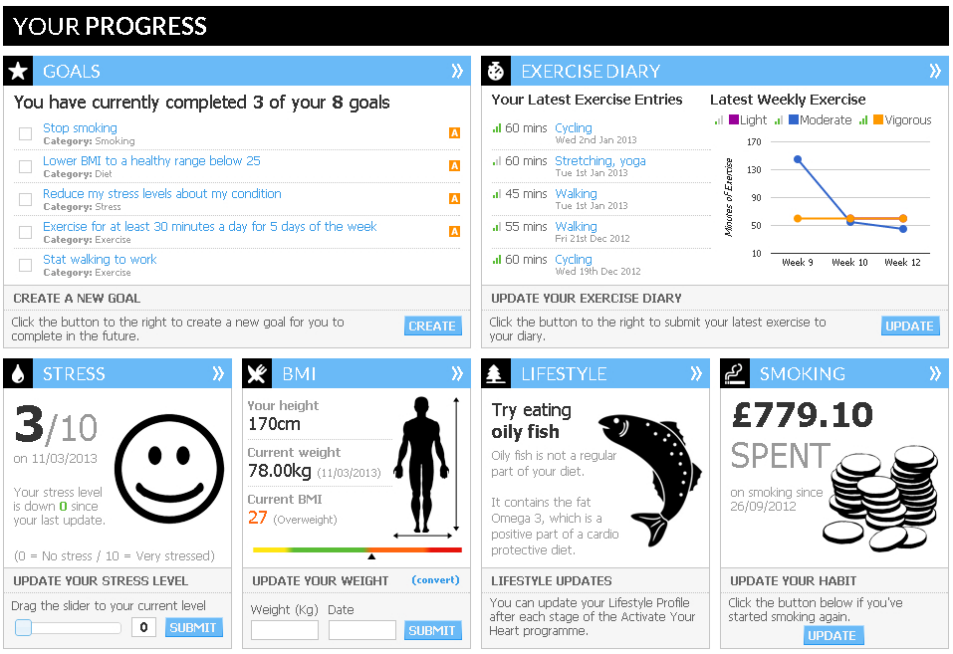
Program goal setting.

At the beginning of the program, each user completed an online form providing information about their medical history and their current cardiac risk factors (Multimedia Appendices 1 and 2). This information was used to set individualized tailored goals focused on exercise (eg, being physically active for 30 minutes 5 times a week), diet (eg, eating more fruit/vegetables and reducing salt intake), emotions (eg, managing stress and other negative emotions), and smoking (eg, reduce cigarette smoking if relevant) (Figure 1).

Compliance to these goals was regularly assessed (using a short set of questions) and feedback on performance provided (Multimedia Appendix 3). Users making progress were congratulated when set goals were achieved. Throughout the program, goals were reset/modified depending on previous performance. As the user progressed through the program, goals set were made increasingly difficult.

Each user also kept an online exercise diary, recording details of their daily exercise (Multimedia Appendix 4). Feedback on the users’ physical activity levels was also provided as they progressed through the program. Users who smoked cigarettes were provided with feedback regarding the amount of money they had spent/saved by smoking/reducing smoking. The program also contained written information about the health consequences of heart disease and a vast amount of information about CHD-related risk factors (exercise, diet, sexual activity, driving, returning to work, hobbies, holidays, benefits, smoking, anxiety, and emotions).

**Figure 2:**
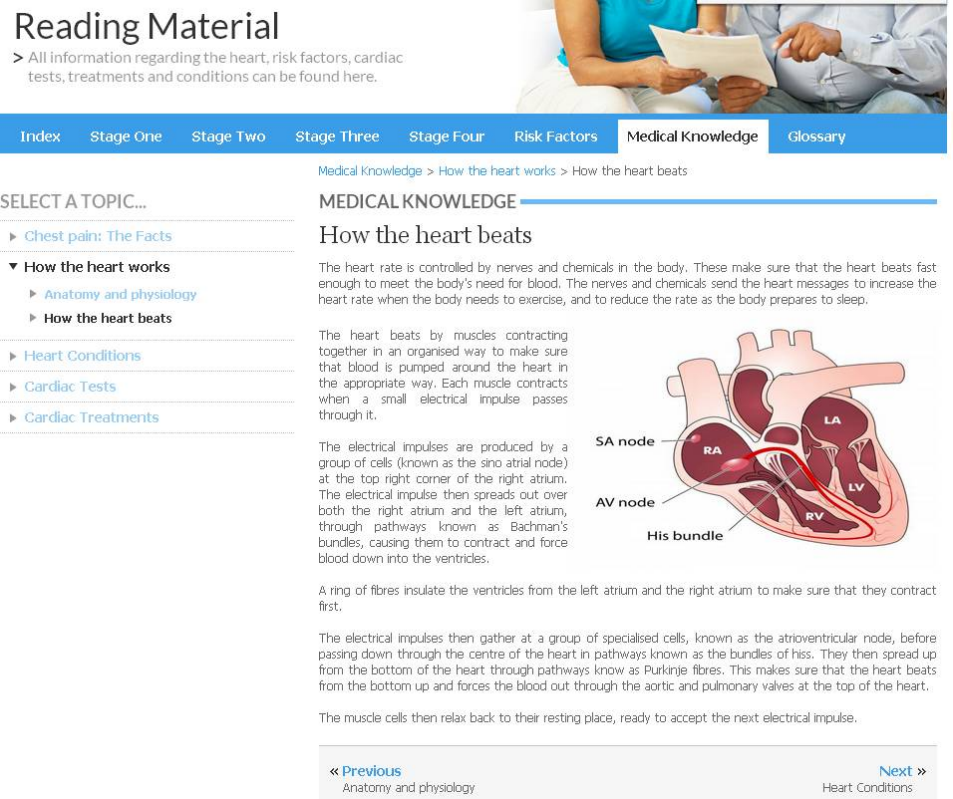
Information about heart disease contained in the program.

In addition, the programme aimed to reduce negative emotions by providing advice about stress/anxiety management skills (see Multimedia Appendix 5). The program also contained information to help users understand heart disease (Figure 2). Program users could initiate contact with cardiac rehabilitation nurses for advice and support via an online email link (see Multimedia Appendix 6) or by joining a scheduled synchronized chat room held on a weekly basis. The cardiac nurses were based at University Hospitals of Leicester. All participants in the intervention group used the program from home and were encouraged to log in to the program 3-4 times per week.

### Control

Participants in the control group continued with treatment as usual from their GP and received no further contact from the researcher until the 6-week follow-up. Usual care in primary care for this population in the United Kingdom constitutes being placed on a CHD register and attending an annual check of risk factor management, usually with a practice nurse.

### Sample Size Calculation

Sample size was based on detecting a significant change in the number of steps walked by participants at the 6-week follow-up. Using previous data, our sample size calculation was based on detecting a difference in means of 3501 steps walked between the intervention and control group [27]. This would require 24 (total 48) participants in each group (with 90% power and .05 significance). We recruited more than this (N=94, 96% more participants) to allow for dropout (often high in studies of Web-based interventions) and to allow for the detection of differences between secondary measures.

### Statistical Methods

Demographic characteristics and baseline measures were compared at baseline using Pearson chi-square tests (categorical variables), independent samples *t* tests (continuous, normally distributed data), and Mann-Whitney *U* tests (nonnormally distributed data). Fisher exact test was used when chi-square test assumptions were violated. Baseline outcome measures in trial completers and trial dropouts were also compared. Change from baseline to follow-up time points in both primary and secondary outcome variables were calculated (follow-up score or value – baseline score or value). The change values in each group were then compared using an independent sample *t* test (normally distributed data) or Mann-Whitney *U* test (nonnormally distributed data). We chose to examine the change in primary/secondary outcome measures at 6-week and 6-month follow-ups and compare this value between groups. This approach to the analysis ensured that all participants’ available data could be used irrespective of study completion level.

All statistical analyses were carried out using SPSS version 22 (IBM Corp, Armonk, NY, USA). Data were analyzed using intention-to-treat analyses; all participants with data available were included in the data analysis according to the group first assigned at randomization regardless of intervention compliance or adherence. Attrition was low; therefore, we did not use any imputation techniques to deal with attrition. Two-tailed findings were reported.

### Ethics

The study protocol gained ethical approval granted by the National Health Service Research Ethics Service (ref: 08/H1210/84) and by Coventry University.

## Results

### Participation Rates

**Figure 3:**
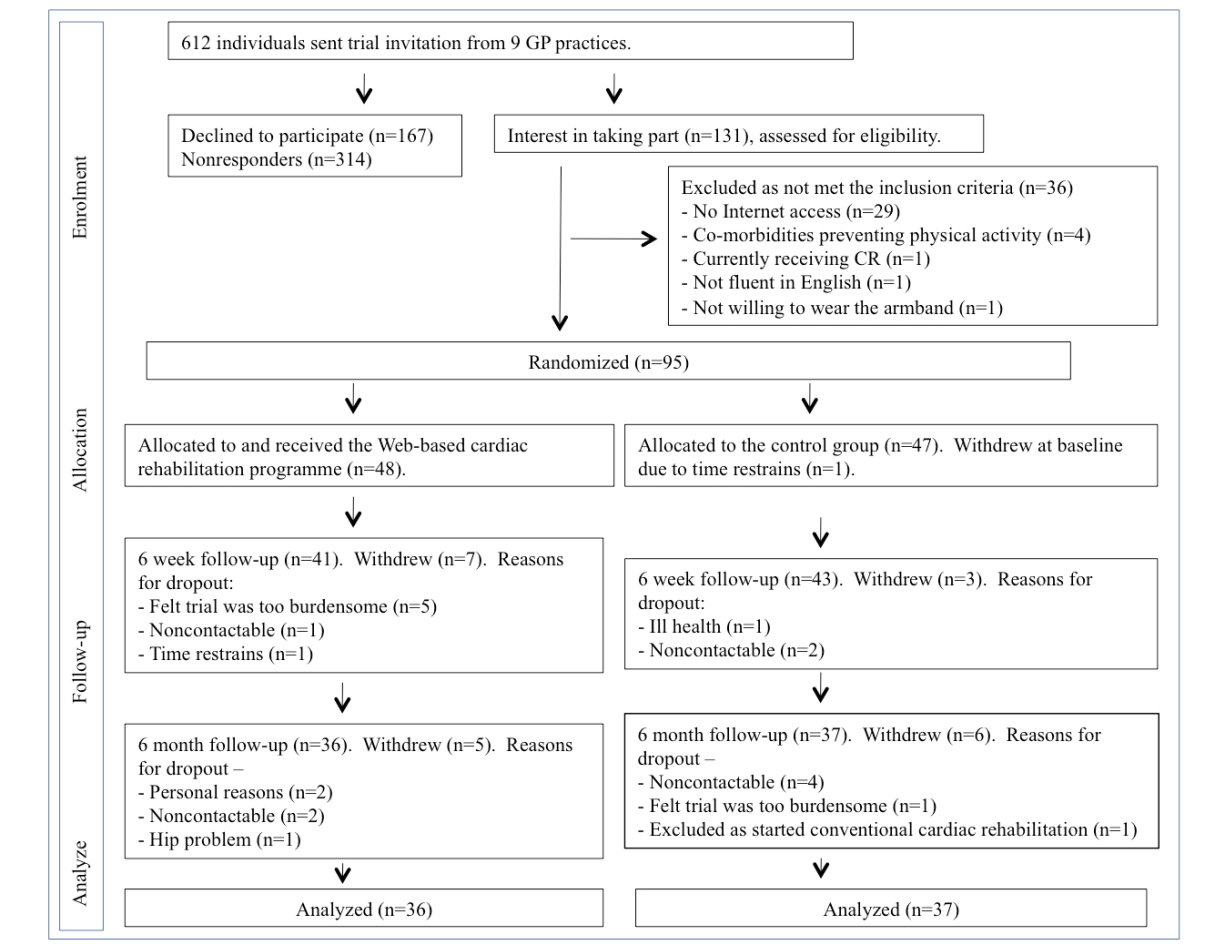
Participant flow through the trial.

A total of 612 patients were invited to take part; 481 (78.6%) declined/did not respond and 131 (21.4%) expressed an interest in the study, of which 95 (15.5%) consented to the trial (Figure 3). A total of 94 participants (99%) completed the baseline measures, 84 (89%) completed the 6-week follow-up (11% attrition), and 73 (78%) completed the 6-month follow-up (22% attrition). At baseline, SBP was higher in those who dropped out (mean 145.19 mm Hg, SD 12.53) compared to those who completed the study (mean 132.95 mm Hg, SD 16.28; *P*=.002). There were no other statistically significant differences between trial completers and trial dropouts in demographic characteristics or baseline outcome measures. Participant flow throughout the trial is shown in Figure 3.

### Demographic Characteristics and Baseline Measures

Participant demographic details are outlined in Table 1. There were no significant differences between the intervention and control group in demographic characteristics or baseline measures.

### Short-Term Intervention Effects

Table 2 outlines baseline and 6-week follow-up values, and change values for all outcomes.

**Table 1:** Demographic characteristics of participants.

Demographic characteristic		Intervention group (n=48)	Control group (n=46)
Age (years), mean (SD)		66.27 (8.35)	66.20 (10.06)
**Gender, n (%)**			
	Male	34 (71)	36 (78)
	Female	14 (29)	10 (22)
**Employment, n (%)**			
	Retired	29 (60)	21 (46)
	Full-time	13 (27)	18 (39)
	Part-time	4 (8)	7 (15)
	Unemployed	2 (4)	0 (0)
**Ethnicity, n (%)**			
	White British	44 (92)	42 (91)
	Other	4 (8)	4 (9)
Years since diagnosis, mean (SD)		7.98 (4.53)	9.44 (5.81)
**Angina treatment, n (%)**			
	Medication only	19 (44)	16 (37)
	Stent(s)	15 (35)	21 (49)
	Coronary artery bypass graft	9 (21)	6 (14)
**Previous cardiac rehabilitation, n (%)**			
	No	34 (76)	35 (81)
	Yes	11 (24)	8 (19)
**Current smoking status, n (%)**			
	No	46 (96)	40 (87)
	Yes	2 (4)	6 (13)

**Table 2:** Short-term intervention effects at baseline (T0) and 6-week follow-up (T1) for the intervention and control groups, including within- and between-group differences (D).

Outcome^a^			Intervention group, mean (SD)				Control group, mean (SD)							
			n^b^	T0	T1	D	n^c^	T0	T1	D	D	*P* ^d^	ES	95% CI
**Physical activity**														
	Daily steps		35	6716 (3060)	7212 (3188)	+497 (2171)	40	6624 (3189)	5763 (2533)	–861 (2534)	1357	.02	0.58	263, 2451
	Daily EE (kcal)		35	1902.47 (392.32)	1946.41 (351.79)	+43.94 (271.90)	40	2055.05 (431.80)	1922.04 (306.47)	–133.01 (302.01)	176.96	.01	0.62	43.93, 309.98
	DSA (min)^f^		35	675.00 (45.00)	671.50 (55.50)	–7.79^e^ (40.14)	40	663.25 (103.25)	672.25 (61.75)	+23.23^e^ (62.78)	–31.01	.01	0.59	–55.01, –7.01
	DMA (min)^f^		35	43.50 (43.00)	48.50 (50.00)	+6.31^e^ (34.37)	40	55.50 (96.25)	47.75 (61.38)	–22.29^e^ (61.34)	28.60	.01	0.58	6.01, 51.20
**Physiological measures**														
	Weight (kg)		41	82.80 (13.49)	82.24 (13.30)	–0.56 (2.00)	42	79.52 (14.36)	79.93 (14.74)	+0.40 (1.71)	–0.97	.02	0.52	–1.78, –0.15
	Body fat (%)		39	38.78 (10.80)	38.36 (11.52)	–0.42 (7.67)	41	36.34 (8.01)	37.01 (7.07)	+0.68 (6.39)	–1.09	.49	0.16	–4.23, 2.04
	SBP (mm Hg)		40	131.35 (15.34)	130.80 (14.70)	–0.55 (12.03)	42	137.55 (16.51)	128.55 (14.88)	–9.00 (12.77)	8.45	.003	0.68	2.99, 13.91
	DBP (mm Hg)		39	72.92 (9.95)	69.00 (9.57)	–3.92 (8.75)	42	72.52 (10.73)	68.52 (9.16)	–4.00 (8.27)	0.08	.97	0.01	–3.69, 3.84
**Diet**														
	Fat score		33	38.76 (8.46)	35.55 (9.18)	–3.21 (7.98)	32	40.88 (11.63)	39.38 (10.38)	–1.50 (11.89)	–1.71	.50	0.17	–6.72, 3.29
	Fiber score		35	36.40 (9.84)	36.51 (8.77)	+0.11 (6.88)	33	35.09 (12.46)	33.79 (12.24)	–1.30 (12.14)	1.42	.55	0.14	–3.33, 6.16
**Psychological**														
	Anxiety score		36	5.61 (3.57)	4.14 (3.50)	–1.47 (3.19)	39	5.51 (3.42)	4.87 (3.73)	–0.64 (2.27)	–0.83	.20	0.30	–2.10, 0.44
	Depression scores^f^		37	3.00 (4.00)	2.00 (2.00)	–0.43^e^ (2.15)	42	2.00 (3.00)	2.00 (4.25)	+0.10^e^ (2.30)	–0.53	.30	0.24	–1.53, 0.48
	Self-efficacy score		37	49.03 (6.55)	51.70 (6.37)	+2.68 (5.92)	39	49.79 (7.56)	49.92 (7.76)	+0.13 (3.49)	2.55	.03	0.52	0.30, 4.79
**MacNew QOL**														
	Emotional score^f^		36	5.89 (1.21)	6.25 (1.04)	+0.31^e^ (0.67)	40	5.96 (1.45)	6.32 (1.21)	+0.04^e^ (0.44)	0.27	.04	0.48	0.01, 0.54
	Physical score^f^		33	6.50 (0.71)	6.50 (0.92)	+0.04^e^ (0.69)	41	6.50 (1.42)	6.58 (1.33)	+0.11^e^ (0.57)	–0.07	.62	0.11	–0.37, 0.22
	Social score^f^		34	6.54 (0.85)	6.73 (0.50)	+0.21^e^ (0.66)	40	6.54 (1.17)	6.62 (1.19)	+0.07^e^ (0.57)	0.14	.34	0.23	–0.15, 0.42
**SAQ** ^g^														
	Physical limitations score		37	64.19 (21.55)	62.16 (25.43)	–2.03 (19.20)	42	63.49 (25.40)	63.69 (27.03)	+0.20 (15.19)	–2.23	.57	0.13	–9.94, 5.49
	Angina stability score^f^		33	42.86 (57.14)	33.33 (66.67)	–9.74^e^ (39.81)	37	42.86 (57.14)	33.33 (66.67)	–9.97^e^ (33.63)	0.23	.98	0.01	–17.29, 17.53
	Angina frequency score		33	43.56 (31.58)	53.79 (30.70)	+10.23 (26.78)	41	44.51 (32.36)	32.93 (28.74)	–11.59 (29.63)	21.81	.002	0.77	8.57, 35.05
	Treatment satisfaction score^f^		35	100.00 (0.00)	100.00 (0.00)	+4.04^e^ (23.38)	36	100.00 (28.57)	100.00 (22.22)	–1.90^e^ (30.52)	5.93	.36	0.22	–6.97, 18.83
	Disease perception score^f^		36	83.33 (33.33)	80.00 (40.00)	+0.97^e^ (20.15)	40	83.33 (39.58)	80.00 (40.00)	–2.13^e^ (17.54)	3.10	.48	0.16	–5.52, 11.71

^a^Daily steps was the primary outcome measure. DBP: diastolic blood pressure; DMA: duration of moderate activity; DSA: duration of sedentary activity; EE: energy expenditure; QOL: quality of life; SAQ: Seattle Angina Questionnaire; SBP: systolic blood pressure.

^b^Number of participants in the intervention group with complete baseline and 6-week follow-up data.

^c^Number of participants in the control group with complete baseline and 6-week follow-up data.

^d^Independent samples *t* test comparing change scores.

^e^The change values were normally distributed; therefore, mean (SD) values reported.

^f^Baseline and 6-week follow-up values were not normally distributed; therefore, median (IQR) values reported.

^g^Higher scores on this questionnaire represent better functioning.

### Primary Outcome Measure

At 6 weeks, the intervention group had greater improvements in step count (+497 steps), whereas the control group had decreased level of steps (–861 steps), yielding an overall medium weight mean effect of 0.58 (95% CI 263-2451, *P*=.02).

### Secondary Outcome Measures

Table 2 outlines the significant improvements in EE (ES=0.62, 95% CI 43.93-309.98, *P*=.01), DSA (ES=0.59, 95% CI –55.01 to –7.01, *P*=.01), DMA (ES=0.58, 95% CI 6.01-51.20, *P*=.01), weight (ES=0.52, 95% CI –1.78 to –0.15, *P*=.02), self-efficacy (ES=0.52, 95% CI 0.30-4.79, *P*=.03), emotional QOL score (ES=0.48, 95% CI 0.01-0.54, *P*=.04), and angina frequency (ES=0.77, 95% CI 8.57-35.05, *P*=.002) in the intervention group compared to the control group at the 6-week follow-up. Unexpectedly, there was also a significantly greater reduction in SBP in the control group compared to the Web-based cardiac rehabilitation group (ES=0.68, 95% CI 2.99-13.91, *P*=.001).

### Medium-Term Intervention Effects

There were significantly lower levels of angina frequency (ES=0.63, 95% CI 1.89-29.41, *P*=.03) and increased social QOL score (ES=0.60, 95% CI 0.05-0.54, *P*=.02) favoring the intervention group at the 6-month follow-up. In contrast, there were no significant medium-term intervention effects in daily steps (ES=0.24, 95% CI–358 to 2324, *P*=.15), daily EE (EE=0.38, 95% CI –35.17 to 250.47, *P*=.14), DSA (ES=0.55, 95% CI 0.190-0.205, *P*=.20), DMA (ES=0.55, 95% CI 0.244-0.261, *P*=.24), weight (ES=0.35, 95% CI –2.46 to 0.34, *P*=.14), body fat percentage (ES=0.00, 95% CI –3.81 to 3.81, *P*>.99), SBP (ES=0.15, 95% CI –4.84 to 9.29, *P*=.53), DBP (ES=0.03, 95% CI –4.80 to 4.29, *P*=.91), fat intake (ES=0.30, 95% CI –6.12 to 1.80, *P*=.28), fiber intake (ES=0.29, 95% CI –2.23 to 8.53, *P*=.25), depression (ES=0.35, 95% CI –2.11 to 0.34, *P*=.15), anxiety (ES=0.47, 95% CI –2.60 to 0.04, *P*=.06), self-efficacy (ES=0.09, 95% CI –2.32 to 3.34, *P*=.72), physical QOL score (ES=0.29, 95% CI –0.11 to 0.43, *P*=.24), emotional QOL score (ES=0.46, 95% CI –0.02 to 0.62, *P*=.06), physical limitations (ES=0.08, 95% CI –7.20 to 10.50, *P*=.71), angina stability (ES=0.13, 95% CI –13.72 to 24.18, *P*=.58), treatment satisfaction (ES=0.08, 95% CI –15.31 to 10.69, *P*=.72), or disease perception (ES=0.17, 95% CI –8.41 to 14.99, *P*=.58). Although there were no significant intervention effects present for many of the outcome measures, it should be acknowledged that at the 6-month follow-up the intervention group showed trends of improved levels of baseline daily steps, EE, DSA, DMA, and weight, whereas the control group declined at the 6-month follow-up.

### Usage of and Adherence to the Rehabilitation Program

Of the 48 intervention group participants, 19 (40%) completed the intervention and 29 (60%) did not progress past stage 3. The mean number of log-ins to the program was 18.68 (SD 13.13, range 1-51), an average of 3 log-ins per week per participant.

## Discussion

### Principal Findings

This study demonstrated daily physical activity improved as identified by step counts (our primary outcome). We also found significant improvements in a range of secondary outcome measures derived from the monitor, most importantly a reduction in sedentary time and an increase in the time spent being moderately active. This change in activity is an important outcome for this study because an important component of the website is to encourage daily exercise, most commonly walking. Although the changes were not significantly better at 6 months, there was a trend for the intervention group to remain improved compared to the control group, of which the effect sizes ranged from small to medium. This is in the absence of continued access to the site or any ongoing support. At the 6-week follow-up, we also observed important changes in weight, self-efficacy, emotional QOL score, and angina symptoms. We also observed significant changes at 6 months in angina symptoms and social QOL score.

### Comparison With Previous Research

The use of technology and telehealth has been described previously in the literature to support individuals with CHD, but most of the studies have used telephone support as the technology [28]. The Internet has been used in a few projects examining a similar type of intervention. Antypas et al [9] recently assessed the effectiveness of an Internet- and mobile phone-based intervention for physical activity in a CHD population and reported increased physical activity levels in the intervention group compared to a control group at the 3-month follow-up; however, the study only had 7 participants at follow-up and measured physical activity using self-reported measures. Southard et al [29] reported on an Internet-based intervention conducted in a mixed population across primary and secondary care; their data suggested that the intervention was effective in some areas, but failed to change levels of self-reported physical activity. Reid et al [8] reported significant group effects in physical activity and QOL following a Web-based physical activity intervention given to patients who had undergone percutaneous coronary revascularization. However, the intervention was not a comprehensive CHD secondary prevention package and targeted physical activity only.

Our current study has demonstrated significant improvements at the 6-week follow-up in walking, DSA, and DMA in comparison to a control group. We also observed improvements in weight, emotional QOL score, self-efficacy, and angina symptoms. At the 6-month follow-up, we were also able to demonstrate lowered angina symptoms, increased social QOL scores, and trends for physical activity to remain improved in the intervention group compared to the control group at the 6-month follow-up. This was in the absence of continued access to the site or any ongoing support because participants did not receive any support between the 6-week and 6-month follow-up assessment. This may not reflect what would happen in practice if this intervention was adopted in the health service.

Previous trials of a manual-based, self-management approach, The Angina Plan, reported significantly increased self-reported physical activity postintervention and at the 6-month follow-up [6,7]. Interestingly, the current study recruited participants with an established diagnosis of angina, whereas previous angina trials recruited those with a new diagnosis, a stage when motivation to adopt a healthier lifestyle may be higher. The Angina Plan is delivered over 12 weeks and comprises an initial in-depth consultation with a trained nurse and close facilitation by the nurse to encourage and discuss progress with agreed patient goals. The current online program was not facilitated in the same way; instead, the contact was initiated by the user via email. One might speculate that this would be a more cost-effective mode of delivery.

The proportion of intervention group participants completing the whole intervention was 40%, and 60% of participants progressed three-quarters of the way through the intervention (up to stage 3). This is comparable with Reid et al [8] who reported 43% of participants completed a Web-based physical activity intervention. Intervention completion rates in both the current study and Reid et al [8] are similar to the completion rate of traditional cardiac rehabilitation. In the United Kingdom between 2011 and 2012, an average of 52% of patients enrolling onto cardiac rehabilitation completed the intervention [4]. Overall, there were also regular website visits with an average of 3 log-ins per week. The mean number of website log-ins was 2 and 4 times per week in Southard et al [29] and Zutz et al [30], respectively.

### Strengths and Limitations

This study evaluated the effects of an Internet-delivered self-managed cardiac rehabilitation program in an angina population with objectively measured physical activity as the primary outcome, a group seldom included within rehabilitation research or rehabilitation services despite current guidelines [31]. The researcher who collected the outcome measures also delivered the intervention. This allows for potential bias because participants with particularly high CHD risk could have unintentionally been encouraged more than other participants, which could have influenced the trial results. In future trials, researchers taking outcome measures should be blinded. In addition, it is necessary to consider measurement reactivity, in which measurement results in changes in the people being measured [32]. Although the study measured physical activity objectively, there still remains the possibility that participants may have adjusted their behavior while the activity monitor was worn.

The physical activity measurement period was 2 days. At the time, 2 days was the recommended monitoring period [33]. For future studies, we would propose wearing the monitor for a longer period, ideally 7 days. The study did not achieve the changes in physical activity that the power calculation was based on. In hindsight, this would appear to be an ambitious target because the power calculation was based on an intervention that was much more intense than the one described here. The data show that the intervention was effective in the short term, and the benefit was sustained in some outcomes at 6-month follow-up in the absence of access to the site or any ongoing support. In the future, we would wish to study the impact of continued access to the site for an extended period compared to best usual care. Due to limitations in funding, we were unable to collect any cost-effectiveness or health care utilization data, which would be desirable in future studies. Additionally, it would be valuable to assess if this intervention has an impact on smoking behavior. The current intervention does comprise a smoking cessation component, although the effect of this component was not examined in the current study because only 2 (4%) and 6 (13%) participants in the intervention and control group, respectively, were smokers at baseline. Future research should examine the intervention’s impact on smoking cessation. The sample recruited in this study was primarily of a White British origin. Although this is not reflective of the general population, it is in-line with the ethnicity of patients currently receiving traditional cardiac rehabilitation as reported in a national audit. Challenges remain to find an acceptable intervention for ethnic minorities [4]. It would also be useful in future studies to compare the outcomes of an angina population using the Web-based rehabilitation program to an angina population receiving traditional rehabilitation.

In terms of the technological advances in health care, the program could also be developed into an application for use on a smartphone, and thereby enable the program to be available via mobile phone technology. Research examining the value of mobile phone-based interventions in increasing physical activity has been evaluated in a meta-analysis conducted by Fanning et al [34] and provides support for interventions using mobile technology to increase physical activity behavior.

### Conclusions

The provision of support for those with angina is poor and these individuals are underrepresented in conventional cardiac rehabilitation programs [4]. An Internet-based approach may offer an alternative self-management approach to either the Angina Plan or cardiac rehabilitation. The program is also likely to offer a lower-cost form of intervention and implementation of a Web-based alternative. This could widen the reach of rehabilitation and effectively increase service capacity. A large, pragmatic trial is required to examine the effectiveness and cost-effectiveness of this intervention when embedded into clinical practice. We would propose to offer access for a longer period of time. There may also be opportunities to explore the value of this intervention as an alternative to conventional rehabilitation for those who have suffered an acute cardiac event; this would help to increase the choice and scope of rehabilitation services.

## Multimedia Appendix 1

Program Registration.

## Multimedia Appendix 2

Program Registration.

## Multimedia Appendix 3

Feedback on user performance.

## Multimedia Appendix 4

Exercise diary.

## Multimedia Appendix 5

Stress management component.

## Multimedia Appendix 6

Communication with cardiac rehabilitation nurses via an online email link; ‘ask the expert’.

## Multimedia Appendix 7

CONSORT-EHEALTH checklist V1.6.2 [35].

## Abbreviations

CHD: coronary heart disease
DMA: duration of moderate activity
DBP: diastolic blood pressure
DSA: duration of sedentary activity
EE: energy expenditure
GP: general practitioner
NIHR: National Institute for Health Research
QOL: quality of life
SAQ: Seattle Angina Questionnaire
SBP: systolic blood pressure

## References

[1] Buckley BS, Simpson CR, McLernon DJ, Murphy Aw, Hannaford Pc (2009). Five year prognosis in patients with angina identified in primary care: incident cohort study. BMJ.

[2] Stewart S, Murphy NF, Murphy N, Walker A, McGuire A, McMurray JJ (2003). The current cost of angina pectoris to the National Health Service in the UK. Heart.

[3] (2008). Cardiac rehabilitation service, Commissioning guide: Implementing NICE guidance.

[4] British Heart Foundation (2013). The National Audit of Cardiac Rehabilitation: Annual Statistical Report 2013.

[5] McGillion M, Arthur H, Victor JC, Watt-Watson J, Cosman T (2008). Effectiveness of Psychoeducational Interventions for Improving Symptoms, Health-Related Quality of Life, and Psychological well Being in Patients with Stable Angina. Curr Cardiol Rev.

[6] Lewin RJ, Furze G, Robinson J, Griffith K, Wiseman S, Pye M, Boyle R (2002). A randomised controlled trial of a self-management plan for patients with newly diagnosed angina. Br J Gen Pract.

[7] Furze G, Cox H, Morton V, Chuang Lh, Lewin Rj, Nelson P, Carty R, Norris H, Patel N, Elton P (2012). Randomized controlled trial of a lay-facilitated angina management programme. J Adv Nurs.

[8] Reid RD, Morrin LI, Beaton LJ, Papadakis S, Kocourek J, McDonnell L, Slovinec D'Angelo ME, Tulloch H, Suskin N, Unsworth K, Blanchard C, Pipe AL (2012). Randomized trial of an internet-based computer-tailored expert system for physical activity in patients with heart disease. Eur J Prev Cardiol.

[9] Antypas K, Wangberg SC (2014). An Internet- and mobile-based tailored intervention to enhance maintenance of physical activity after cardiac rehabilitation: short-term results of a randomized controlled trial. J Med Internet Res.

[10] van den Berg MH, Schoones JW, Vliet Vlieland TP (2007). Internet-based physical activity interventions: a systematic review of the literature. J Med Internet Res.

[11] Wijsman CA, Westendorp RG, Verhagen EA, Catt M, Slagboom PE, de Craen AJ, Broekhuizen K, van Mechelen W, van Heemst D, van der Ouderaa F, Mooijaart SP (2013). Effects of a web-based intervention on physical activity and metabolism in older adults: randomized controlled trial. J Med Internet Res.

[12] Richardson CR, Buis LR, Janney AW, Goodrich De, Sen A, Hess Ml, Mehari Ks, Fortlage La, Resnick Pj, Zikmund-Fisher Bj, Strecher Vj, Piette Jd (2010). An online community improves adherence in an internet-mediated walking program. Part 1: results of a randomized controlled trial. J Med Internet Res.

[13] Jakicic JM, Marcus M, Gallagher KI, Randall C, Thomas E, Goss FL, Robertson RJ (2004). Evaluation of the SenseWear Pro Armband to assess energy expenditure during exercise. Med Sci Sports Exerc.

[14] Dwyer TJ, Alison JA, McKeough ZJ, Elkins MR, Bye PT (2009). Evaluation of the SenseWear activity monitor during exercise in cystic fibrosis and in health. Respir Med.

[15] Hill K, Dolmage TE, Woon L, Goldstein R, Brooks D (2010). Measurement properties of the SenseWear armband in adults with chronic obstructive pulmonary disease. Thorax.

[16] Roe L, Strong C, Whiteside C, Neil A, Mant D (1994). Dietary intervention in primary care: validity of the DINE method for diet assessment. Fam Pract.

[17] Zigmond AS, Snaith RP (1983). The hospital anxiety and depression scale. Acta Psychiatr Scand.

[18] Bjelland I, Dahl AA, Haug TT, Neckelmann D (2002). The validity of the Hospital Anxiety and Depression Scale. An updated literature review. J Psychosom Res.

[19] De Smedt D, Clays E, Doyle F, Kotseva K, Prugger C, Pająk A, Jennings C, Wood D, De Bacquer D, EUROASPIRE Study Group (2013). Validity and reliability of three commonly used quality of life measures in a large European population of coronary heart disease patients. Int J Cardiol.

[20] Schwarzer R, Jerusalem M, Weinman J, Wright S, Johnston M (1995). Causal and control beliefs. Measures in Health Psychology: A User’s Portfolio.

[21] Schwarzer R, Fuchs R (1996). Self-efficacy and health behaviours. Predicting Health Behaviour: Research and Practice with Social Cognition Models.

[22] Höfer S, Lim L, Guyatt G, Oldridge N (2004). The MacNew Heart Disease health-related quality of life instrument: a summary. Health Qual Life Outcomes.

[23] Spertus JA, Winder JA, Dewhurst TA, Deyo RA, Prodzinski J, McDonell M, Fihn SD (1995). Development and evaluation of the Seattle Angina Questionnaire: a new functional status measure for coronary artery disease. J Am Coll Cardiol.

[24] Höfer S, Saleem A, Stone J, Thomas R, Tulloch H, Oldridge N (2012). The MacNew Heart Disease Health-Related Quality of Life Questionnaire in patients with angina and patients with ischemic heart failure. Value Health.

[25] Activate Your Heart: Online Cardiac Rehabilitation Programme.

[26] Michie S, Richardson M, Johnston M, Abraham C, Francis J, Hardeman W, Eccles Mp, Cane J, Wood Ce (2013). The behavior change technique taxonomy (v1) of 93 hierarchically clustered techniques: building an international consensus for the reporting of behavior change interventions. Ann Behav Med.

[27] Tudor-Locke C, Bell RC, Myers AM, Harris SB, Ecclestone NA, Lauzon N, Rodger NW (2004). Controlled outcome evaluation of the First Step Program: a daily physical activity intervention for individuals with type II diabetes. Int J Obes Relat Metab Disord.

[28] Neubeck L, Ascanio R, Bauman A, Briffa T, Clark AM, Freedman B, Redfern J (2011). Planning locally relevant Internet programs for secondary prevention of cardiovascular disease. Eur J Cardiovasc Nurs.

[29] Southard BH, Southard DR, Nuckolls J (2003). Clinical trial of an Internet-based case management system for secondary prevention of heart disease. J Cardiopulm Rehabil.

[30] Zutz A, Ignaszewski A, Bates J, Lear SA (2007). Utilization of the internet to deliver cardiac rehabilitation at a distance: a pilot study. Telemed J E Health.

[31] National Institute for Health and Care Excellence.

[32] French DP, Sutton S (2010). Reactivity of measurement in health psychology: how much of a problem is it? What can be done about it?. Br J Health Psychol.

[33] Tudor-Locke CE, Myers AM (2001). Methodological considerations for researchers and practitioners using pedometers to measure physical (ambulatory) activity. Res Q Exerc Sport.

[34] Fanning J, Mullen SP, McAuley E (2012). Increasing physical activity with mobile devices: a meta-analysis. J Med Internet Res.

[35] Eysenbach G, CONSORT-EHEALTH Group (2011). CONSORT-EHEALTH: improving and standardizing evaluation reports of Web-based and mobile health interventions. J Med Internet Res.

