# Supplementary material for: A Web-Based Program Improves Physical Activity Outcomes in a Primary Care Angina Population: Randomized Controlled Trial
Source: J Med Internet Res. 2014 Sep 12;16(9):e186. doi: 10.2196/jmir.3340 (PMC4180351; doi:10.2196/jmir.3340)
Supplement: Supplementary file 1 [file jmir_v16i9e186_app1.pdf]

ACTIVATE **YOUR** HEART

## NEW ACCOUNT SETUP

SECTION **ONE**

### Personal Information

- i. Account Details
- ii. Contact Details
- iii. About Yourself

SECTION **TWO**

### Risk Factors

- i. Exercise
- ii. Diet
- iii. Stress
- iv. Smoking
- v. Family History

SECTION **THREE**

### Medical Information

SECTION **ONE** Personal Information

Please take a few moments to complete this registration page. Your responses will help us to tailor the Activate Your Heart programme to best meet your needs. Your responses will be kept confidential.

### ii. CONTACT DETAILS

**1** Title

Select Your Title

**2** First Name

**3** Surname / Family Name

**4** Email Address
