# Supplementary material for: A Web-Based Program Improves Physical Activity Outcomes in a Primary Care Angina Population: Randomized Controlled Trial
Source: J Med Internet Res. 2014 Sep 12;16(9):e186. doi: 10.2196/jmir.3340 (PMC4180351; doi:10.2196/jmir.3340)
Supplement: Supplementary file 2 [file jmir_v16i9e186_app2.pdf]

SECTION ONE
Personal Information
i. Account Details
ii. Contact Details
iii. About Yourself

SECTION TWO
Risk Factors
i. Exercise
ii. Diet
iii. Stress
iv. Smoking
v. Family History

SECTION THREE
Medical Information
i. Current Condition
ii. Previous & Other Conditions
iii. Additional Tests

SECTION FOUR
Knowledge Check
i. Part One
ii. Part Two
iii. Part Three
iv. Results

NEW ACCOUNT SETUP

SECTION FOUR Knowledge Check

The following questionnaire has been designed to assess your current level of understanding about CHD. Please select either 'TRUE', 'FALSE' or 'DON'T KNOW' for each statement. You will be asked to complete this questionnaire again at the end of the programme.

Tick the box which you think is the correct answer

i. PART ONE (Q 1-5 of 13)

**1**	**Coronary Heart Disease (CHD)**	True	False	Don't Know
a	CHD is the world's leading cause of death	✓		
b	Women do not tend to suffer from CHD		✓	
c	CHD is unusual in people less than 40 years old			
d	CHD is where one or more of the coronary arteries have narrowed			✓
e	CHD is a curable disease		✓	

**2**	**Risk factors for Coronary Heart Disease (CHD):**	True	False	Don't Know
a	There is no link between prolonged stress and CHD		✓	
b	A BMI of 25 or higher is associated with an increased risk for CHD	✓		
c	Raised cholesterol levels increase the risk of CHD	✓		
d	CHD can be inherited		✓	
e	The risk of CHD increases with age			✓
