# Supplementary material for: A Web-Based Program Improves Physical Activity Outcomes in a Primary Care Angina Population: Randomized Controlled Trial
Source: J Med Internet Res. 2014 Sep 12;16(9):e186. doi: 10.2196/jmir.3340 (PMC4180351; doi:10.2196/jmir.3340)
Supplement: Supplementary file 3 [file jmir_v16i9e186_app3.pdf]

GOALS

YOUR GOALS

You have currently completed **3** of your **8** goals

TICK TO MARK AS COMPLETE

✔	Go to the gym<br>**Category:** Exercise | **Added:** Wed 13 Jun | **Importance:** 10	Progress (100%)
☐	Reduce smoking<br>**Category:** Smoking | **Added:** Tue 19 Jun | **Importance:** 10	Progress (30%)
