# Supplementary material for: A Web-Based Program Improves Physical Activity Outcomes in a Primary Care Angina Population: Randomized Controlled Trial
Source: J Med Internet Res. 2014 Sep 12;16(9):e186. doi: 10.2196/jmir.3340 (PMC4180351; doi:10.2196/jmir.3340)
Supplement: Supplementary file 4 [file jmir_v16i9e186_app4.pdf]

EXERCISE DIARY

ADD ENTRY

STAGE THREE - EXERCISE TASK

"Complete Task: 30 minutes (continuous) moderate exercise a day for 5 days over a 7 day period"

You have completed this task

EXERCISE SUMMARY

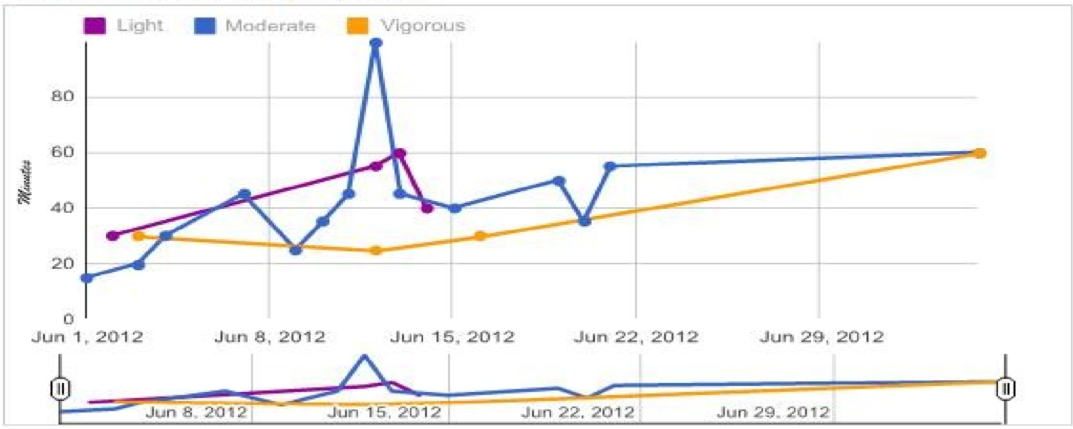

EXERCISE DIARY ENTRIES

DATE	TIME	INTENSITY	ACTIVITY	DIFFICULTY
Thu 5 Jul	60 mins	Moderate	Walking	3
Thu 5 Jul	60 mins	Vigorous	Football	7
Thu 21 Jun	55 mins	Moderate	Walking	5
Wed 20 Jun	35 mins	Moderate	Walking	2
Tue 19 Jun	50 mins	Moderate	Walking	6

FILTER ENTRIES

Activity Type

- Walking
- Stretching, yoga
- Walking
- Cycling, stationary
