# Supplementary material for: A Web-Based Program Improves Physical Activity Outcomes in a Primary Care Angina Population: Randomized Controlled Trial
Source: J Med Internet Res. 2014 Sep 12;16(9):e186. doi: 10.2196/jmir.3340 (PMC4180351; doi:10.2196/jmir.3340)
Supplement: Supplementary file 5 [file jmir_v16i9e186_app5.pdf]

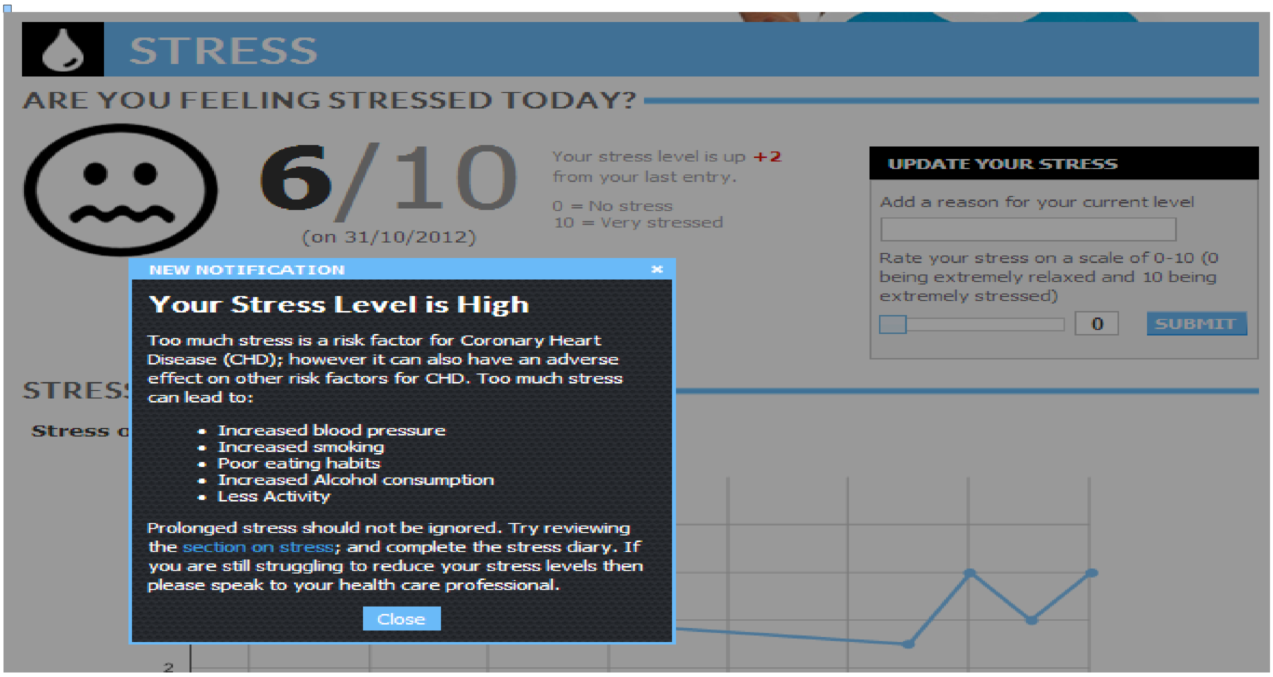
STRESS
ARE YOU FEELING STRESSED TODAY?
6/10
(on 31/10/2012)
Your stress level is up +2 from your last entry.
0 = No stress
10 = Very stressed
UPDATE YOUR STRESS
Add a reason for your current level
Rate your stress on a scale of 0-10 (0 being extremely relaxed and 10 being extremely stressed)
0
SUBMIT
NEW NOTIFICATION
Your Stress Level is High
Too much stress is a risk factor for Coronary Heart Disease (CHD); however it can also have an adverse effect on other risk factors for CHD. Too much stress can lead to:
Increased blood pressure
Increased smoking
Poor eating habits
Increased Alcohol consumption
Less Activity
Prolonged stress should not be ignored. Try reviewing the section on stress; and complete the stress diary. If you are still struggling to reduce your stress levels then please speak to your health care professional.
Close
