# Supplementary material for: A Web-Based Program Improves Physical Activity Outcomes in a Primary Care Angina Population: Randomized Controlled Trial
Source: J Med Internet Res. 2014 Sep 12;16(9):e186. doi: 10.2196/jmir.3340 (PMC4180351; doi:10.2196/jmir.3340)
Supplement: Supplementary file 6 [file jmir_v16i9e186_app6.pdf]

Ask the Expert

> This is a private messaging facility where you can get advice from a healthcare professional.

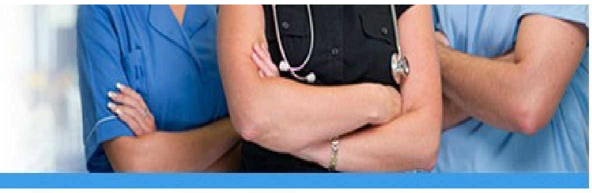

WRITE A NEW MESSAGE

SEND
