# Supplementary material for: A Web-Based Program Improves Physical Activity Outcomes in a Primary Care Angina Population: Randomized Controlled Trial
Source: J Med Internet Res. 2014 Sep 12;16(9):e186. doi: 10.2196/jmir.3340 (PMC4180351; doi:10.2196/jmir.3340)
Supplement: Supplementary file 7 [file jmir_v16i9e186_app7.pdf]

CONSORT-EHEALTH Checklist V1.6.2 Report

(based on CONSORT-EHEALTH V1.6), available at [http://tinyurl.com/consort-ehealth-v1-6].

3340

Date completed

6/17/2014 12:44:44

by

Reena Devi

A Web-based Programme Improves Physical Activity Outcomes in a Primary Care Angina Population: A Randomised Controlled Trial.

TITLE

1a-i) Identify the mode of delivery in the title

"A Web-based Programme Improves Physical Activity Outcomes in a Primary Care Angina Population: A Randomised Controlled Trial"

1a-ii) Non-web-based components or important co-interventions in title

This item is not applicable for our study as there were no non-web-based components in our intervention.

1a-iii) Primary condition or target group in the title

"A Web-based Programme Improves Physical Activity Outcomes in a Primary Care Angina Population: A Randomised Controlled Trial"

ABSTRACT

1b-i) Key features/functionalities/components of the intervention and comparator in the METHODS section of the ABSTRACT

"Intervention group participants were offered a 6 week web-based rehabilitation programme ('ActivateYourHeart'). The programme was introduced during a face-to-face appointment, and then delivered via the internet (no further face-to-face contact). The programme contained information about the secondary prevention of coronary heart disease and set each user goals around physical activity, diet, managing emotions, and smoking. Performance against goals was reviewed throughout the programme and goals were then re-set/modified. Participants completed an online exercise diary and communicated with rehabilitation specialists through an email link/synchronised chat room."
The comparator is also described in the abstract and is as follows: "Participants in the control group continued with GP treatment as usual, which consisted of being placed on a CHD register and attending an annual check."

1b-ii) Level of human involvement in the METHODS section of the ABSTRACT

Yes, we mention that there was opportunity to communicate with the rehabilitation specialist through an email or synchronised chat room link.

"Participants completed an online exercise diary and communicated with rehabilitation specialists through an email link/synchronised chat room".

1b-iii) Open vs. closed, web-based (self-assessment) vs. face-to-face assessments in the METHODS section of the ABSTRACT

Participants were recruited offline: "We conducted a randomised controlled trial, recruiting those diagnosed with angina from General Practitioners (GPs) in primary care to an intervention or control group".

A description of self-assessed questionnaire outcomes have been included: "Self-assessed questionnaire outcomes included fat/fibre intake, anxiety/depression, self-efficacy, and quality-of-life (QOL)".

1b-iv) RESULTS section in abstract must contain use data

This information has been added: "Ninety-four participants were recruited and randomised to the intervention (n=48) or to the usual care (n=46) group, 84 and 73 participants completed the 6-week and 6-month follow-up respectively. The mean (SD, range) number of logins to the programme was 18.68 (SD: 13.13, range 1-51), an average of 3 logins per week per participant. Change in daily steps walked at the 6-week follow-up was +497 (2171) in the intervention group and -861 (2534) in the control group (P=.02, 95% CI: 263:2451). Significant intervention effects were observed at the 6-week follow-up in EE (+43.94kcal, P =.01, 95% CI: 43.93:309.98), DSA (-7.79 minutes, P=.01, 95% CI: -55.01:-7.01), DMA (+6.31 minutes, P=.01, 95% CI: 6.01: 51.20), weight (-0.56kgs, P=.02, 95% CI: -1.78:-0.15), self-efficacy (P=.03, 95% CI: 0.30:4.79), emotional QOL score (P=.04, 95% CI: 0.01:0.54) and angina frequency (P=.002, 95% CI: 8.57:35.05). Significant benefits in angina frequency (P=.026, 95% CI: 1.89:29.41) and social QOL score (P=.018, 95% CI: 0.05:0.54) were also observed at the 6-month follow-up".

1b-v) CONCLUSIONS/DISCUSSION in abstract for negative trials

Not relevant for this study.

INTRODUCTION

2a-i) Problem and the type of system/solution

The problem: "The impact of angina is significant to both the individual [2] and to the health service [3]. Cardiac rehabilitation is recommended for individuals with angina in many international guidelines [4], but capacity to accommodate these individuals is limited and those with a recent cardiac event take priority. Recent data suggests that angina patients constitute only 4% of the referrals to rehabilitation, and almost 20% of programmes do not accept those with angina onto their programme [5]".

The solution: "We have developed an interactive, password protected website specifically for individuals with CHD, which is a comprehensive educational package that aims to improve health behaviours related to CHD. The internet allows for the delivery of a standard intervention that is not geographically or time restrained. It is intended that this intervention could be offered to those not routinely included within traditional cardiac rehabilitation such as those with stable angina."

2a-ii) Scientific background, rationale: What is known about the (type of) system

"The impact of angina is significant to both the individual [2] and to the health service [3]. Cardiac rehabilitation is recommended for individuals with angina in many international guidelines [4], but capacity to accommodate these individuals is limited and those with a recent cardiac event take priority. Recent data suggests that angina patients constitute only 4% of the referrals to rehabilitation, and almost 20% of programmes do not accept those with angina onto their programme [5].

Arguably there is a broad spectrum of interventions that may constitute a rehabilitation programme, from fully supervised sessions to more remote home-based services. It is largely these self-directed home based programmes that have been tested in the angina population. A meta analysis of 7 trials [6] demonstrated that psychoeducational interventions delivered via a trained professional significantly reduced medication use, physical limitations, and disease perception in angina populations. In addition the angina population have been considered previously with a manual based approach: The Angina Plan [7,8], however this has not been widely adopted [5]. A small number of trials have studied the effectiveness of secondary prevention interventions for Coronary Heart Disease (CHD) delivered via the internet. A recent Canadian study [9] evaluated a 6 month web-based physical activity programme for patients who had undergone percutaneous coronary revascularisation. The study did not report baseline scores but the authors reported higher levels of physical activity in the intervention group compared to the control group. The change in physical activity was reported from the 6 to the 12 month follow up in the intervention group, this was significant compared to the control group. Recently a study conducted in Norway assessed the effectiveness of an internet and mobile phone based intervention for physical activity as an extension of face to face cardiac rehabilitation [10]. The study demonstrated significantly higher physical activity levels in the intervention group compared to a control group at a 3 month follow up. The study is however somewhat limited by the small sample size at follow up (n=7) and the self-reported measure of physical activity. The value of web-based interventions and physical activity promotion has also been investigated by Van den Berg et al in a systematic review [11]. Van den Berg et al reviewed 10 articles and reported online interventions are effective in improving physical activity levels [11]. This review emphasised the need to measure physical activity using objective measures. Positive findings have been reported from research measuring physical activity objectively when evaluating web-based physical activity interventions [12,13].

We have developed an interactive, password protected website specifically for individuals with CHD, which is a comprehensive educational package that aims to improve health behaviours related to CHD. The internet allows for the delivery of a standard intervention that is not geographically or time restrained. It is intended that this intervention could be offered to those not routinely included within traditional cardiac rehabilitation such as those with stable angina. The purpose of this study was to assess the clinical effectiveness of this independent internet delivered self managed 'rehabilitation' programme in a population with chronic stable angina in a primary care setting. As we were studying the efficacy of a novel intervention for which we had only limited previous data, our primary hypothesis was non-directional and was 'users of a web-based cardiac rehabilitation programme would alter their coronary risk factors compared to those receiving treatment as usual (control group)'. Two-tailed statistics tests were therefore used as in theory the intervention could have had a negative or a positive effect."

METHODS

3a) CONSORT: Description of trial design (such as parallel, factorial) including allocation ratio

"The purpose of this study was to assess the clinical effectiveness of this independent internet delivered self managed 'rehabilitation' programme in a population with chronic stable angina in a primary care setting. As we were studying the efficacy of a novel intervention for which we had only limited previous data, our primary hypothesis was non-directional and was 'users of a web-based cardiac rehabilitation programme would alter their coronary risk factors compared to those receiving treatment as usual (control group)'. Two-tailed statistics tests were therefore used as in theory the intervention could have had a negative or a positive effect".

3b) CONSORT: Important changes to methods after trial commencement (such as eligibility criteria), with reasons

This item is not relevant for this study.

3b-i) Bug fixes, Downtimes, Content Changes

This item is not relevant for this study.

4a) CONSORT: Eligibility criteria for participants

"Individuals were invited to participate if they had a confirmed diagnosis of stable angina, able to read and speak fluent English, had regular access to the internet, were computer literate, and had not had conventional cardiac rehabilitation within the previous year. Individuals were excluded if they had unstable angina, significant cardiac arrhythmia, any co-morbidities preventing physical activity or were severely anxious/ depressed. Severely anxious/depressed patients were excluded by eliminating anyone with a history of being prescribed medication for either anxiety or depression"

4a-i) Computer / Internet literacy

"Individuals were invited to participate if they had a confirmed diagnosis of stable angina, able to read and speak fluent English, had regular access to the internet, were computer literate, and had not had conventional cardiac rehabilitation within the previous year."

4a-ii) Open vs. closed, web-based vs. face-to-face assessments:

"Participants were recruited offline from 9 primary care General Practitioners (GP) in one region of England."

"Those in the web-based cardiac rehabilitation group received a face to face introductory session from the researcher (RD). This involved registering the individual, creating a unique username/password and demonstrating how to use the programme."

4a-iii) Information giving during recruitment

"During the home visit the researcher explained the study details, took participant consent."

4b) CONSORT: Settings and locations where the data were collected

"During the home visit the researcher explained the study details, took participant consent, and carried out the physical baseline outcome measures (weight, blood pressure, and body fat percentage)."

4b-i) Report if outcomes were (self-)assessed through online questionnaires

"During this initial meeting participants were also given an accelerometer and a questionnaire pack. Each participant was instructed to wear the monitor for 2 week days (12hours per day) and to complete the questionnaires (paper-based questionnaires). This initial meeting lasted approximately 40 minutes."

4b-ii) Report how institutional affiliations are displayed

This has not been described in the manuscript. Our web-based intervention describes the University Hospitals of Leicester NHS Trust affiliation, it is unlikely that this will have created bias within the sample.

5) CONSORT: Describe the interventions for each group with sufficient details to allow replication, including how and when they were actually administered

5-i) Mention names, credential, affiliations of the developers, sponsors, and owners

"The programme was developed at the University Hospitals of Leicester NHS Trust and co-produced with healthcare professionals, a software development team (HARK2), and a group of patients/members of the public."

"Conflicts of Interest: The only study team member related to the intervention being evaluated is SS who participated in the design of the intervention evaluated in this manuscript. SS derives no financial benefit from the development of the intervention."

5-ii) Describe the history/development process

"The programme was developed at the University Hospitals of Leicester NHS Trust and co-produced with healthcare professionals, a software development team (HARK2), and a group of patients/members of the public. Development of the site was an interactive and iterative process, involving patients providing input and feedback on different versions of the website, feeding back on website content, layout, visual features, and ease of website navigation."

5-iii) Revisions and updating

No revisions/updates were made during the evaluation process.

5-iv) Quality assurance methods

Quality assurance methods were not used in this study.

5-v) Ensure replicability by publishing the source code, and/or providing screenshots/screen-capture video, and/or providing flowcharts of the algorithms used

Screenshots of the intervention have been provided in the appendix.

5-vi) Digital preservation

"The intervention was delivered via the internet, and called 'ActivateYourHeart'(www.activateyourheart.org.uk [26]), a secure and password protected site designed for participants to use at home"

We have also archived this reference, and the link can be found in the references.

5-vii) Access

"The intervention was delivered via the internet, and called 'ActivateYourHeart'(www.activateyourheart.org.uk [26]), a secure and password protected site designed for participants to use at home."

"Participants were not paid to take part in this trial"

5-viii) Mode of delivery, features/functionalities/components of the intervention and comparator, and the theoretical framework

"The intervention was delivered via the internet, and called 'ActivateYourHeart'(www.activateyourheart.org.uk [26]), a secure and password protected site designed for participants to use at home. The programme was developed at the University Hospitals of Leicester NHS Trust and co-produced with healthcare professionals, a software development team (HARK2), and a group of patients/members of the public. Development of the site was an interactive and iterative process, involving patients providing input and feedback on different versions of the website, feeding back on website content, layout, visual features, and ease of website navigation. The programme aimed to improve patients' cardiac risk profile within 4 stages, designed to complete within 6 weeks. The intervention used the following behaviour change techniques [27]; 'setting/reviewing behavioural goals', 'self-monitoring', 'feedback on behaviour', 'graded tasks', 'social reward', 'providing information about health consequences', and 'reducing negative emotions'. At the beginning of the programme each user completed an online form providing information about their medical history and their current cardiac risk factors (multimedia appendix 1a and 1b). This information was used to set individualised tailored goals focused on exercise (e.g. being physically active for 30 minutes 5 times a week), diet (e.g. eating more fruit/vegetables and reducing salt intake), emotions (managing stress and other negative emotions) and smoking (reduce cigarette smoking if relevant) (figure 1). Compliance to these goals was regularly assessed (using a short set of questions) and then feedback on performance provided (multimedia appendix 1c). Users making progress were congratulated when set goals were achieved. Throughout the programme goals were re-set/modified depending on previous performance. As the user progressed through the programme goals set were made increasingly difficult. Each user also kept an online 'exercise diary', recording details of their daily exercise (multimedia appendix 1d), feedback on the users' physical activity levels was also provided as they progressed through the programme. Users who smoked cigarettes were also provided with feedback regarding the amount of money they had spent/saved by smoking/reducing smoking. The programme also contained written information about the health consequences of heart disease, and a vast amount of information about CHD related risk factors (exercise, diet, sexual activity, driving, returing to work, hobbies, holidays, benefits, smoking, anxiety and emotions). The programme also aimed to reduce negative emotions through providing advice about stress/anxiety management skills (see multimedia appendix 1e). The programme also contained information to help users understand what heart disease is (figure 2). Programme users could initiate contact with cardiac rehabilitation nurses for advice and support via an online email link (see multimedia appendix 1f) or by joining a scheduled synchronised chat room held on a weekly basis. The cardiac nurses were based at University Hospitals of Leicester. All participants in the intervention group used the programme from home and were encouraged to log into the programme 3-4 times per week."

5-ix) Describe use parameters

"Intervention group participants were told to log into the programme daily in order to record their daily physical activity diary."

5-x) Clarify the level of human involvement

"Programme users could initiate contact with cardiac rehabilitation nurses for advice and support via an online email link (see multimedia appendix 1f) or by joining a scheduled synchronised chat room held on a weekly basis."

5-xi) Report any prompts/reminders used

This item has not been described as prompts were not used in this study.

5-xii) Describe any co-interventions (incl. training/support)

"Those in the web-based cardiac rehabilitation group received a face to face introductory session from the researcher (RD). This involved registering the individual, creating a unique username/password and demonstrating how to use the programme."

6a) CONSORT: Completely defined pre-specified primary and secondary outcome measures, including how and when they were assessed

Yes.

6a-i) Online questionnaires: describe if they were validated for online use and apply CHERRIES items to describe how the questionnaires were designed/deployed

No, all questionnaires were paper-based in this trial.

"Each participant was instructed to wear the monitor for 2 week days (12 hours per day) and to complete the questionnaires (paper-based questionnaires)."

6a-ii) Describe whether and how "use" (including intensity of use/dosage) was defined/measured/monitored

"In the intervention group we also monitored the number of 'logins' to the online programme, this information is available from the admin side of the intervention"

6a-iii) Describe whether, how, and when qualitative feedback from participants was obtained

Qualitative feedback was collected, we intend to publish these findings separately.

6b) CONSORT: Any changes to trial outcomes after the trial commenced, with reasons

There were no changes to the trial outcomes after the trial commenced.

7a) CONSORT: How sample size was determined

7a-i) Describe whether and how expected attrition was taken into account when calculating the sample size

"This was based on detecting a significant change in the number of steps walked by participants at the 6-week follow-up. Using previous data our sample size calculation was based on detecting a difference in means of 3501 steps walked between the intervention and control group [28]. This would require 24 (total 48) participants in each group (with 90% power, and 0.05 significance). We recruited more than this (n=94, 96%) to allow for drop-out (often high in studies of web-based interventions) and to allow for the detection of differences between secondary measures."

7b) CONSORT: When applicable, explanation of any interim analyses and stopping guidelines

We conducted interim analyses, however this analysis is not reported here.

8a) CONSORT: Method used to generate the random allocation sequence

"A computerised block randomisation list was produced by our departmental statistician."

8b) CONSORT: Type of randomisation; details of any restriction (such as blocking and block size)

"A computerised block randomisation list was produced by our departmental statistician."

9) CONSORT: Mechanism used to implement the random allocation sequence (such as sequentially numbered containers), describing any steps taken to conceal the sequence until interventions were assigned

"Allocation concealment was achieved by sequentially numbered sealed envelopes, opened after baseline data collection for each participant by the researcher carrying out the field work (RD)."

10) CONSORT: Who generated the random allocation sequence, who enrolled participants, and who assigned participants to interventions

Generation of random allocation: "A computerised block randomisation list was produced by our departmental statistician."

Enrolment of participants: "During the home visit the researcher (RD) explained the study details, took participant consent, and carried out the physical baseline outcome measures (weight, blood pressure, and body fat percentage)."

11a) CONSORT: Blinding - If done, who was blinded after assignment to interventions (for example, participants, care providers, those assessing outcomes) and how

11a-i) Specify who was blinded, and who wasn't

No one was blinded in this trial.

11a-ii) Discuss e.g., whether participants knew which intervention was the "intervention of interest" and which one was the "comparator"

"During the home visit the researcher (RD) explained that the purpose of the study was to investigate the effectiveness of a web-based intervention, described study details."

11b) CONSORT: If relevant, description of the similarity of interventions

Not relevant.

12a) CONSORT: Statistical methods used to compare groups for primary and secondary outcomes

"Statistical Methods: Demographic characteristics and baseline measures were compared at baseline using Pearsons chi-square test (categorical variables), independent samples t-tests (continuous, normally distributed data) and Mann Whitney U tests (non-normally distributed). Fishers Exact test was used when chi-square test assumptions were violated. 'Trial completers' and 'trial dropouts' characteristics and baseline outcome measures were also compared. Change from baseline to follow-up time points in both primary and secondary outcome variables was calculated (follow-up score/value minus baseline score/value). The change values in each group were then compared using an independent sample t test (normally distributed data) or Mann-Whitney U test (non-normally distributed data). We chose to examine the change in primary/secondary outcome measures at the 6 week and 6 month follow up and compare this value between groups. This approach to the analysis ensured that all participants' available data could be used irrespective of study completion level."

12a-i) Imputation techniques to deal with attrition / missing values

"Attrition was low and therefore we didn't use any imputation techniques to deal with attrition."

12b) CONSORT: Methods for additional analyses, such as subgroup analyses and adjusted analyses

We did not carry out any additional analyses or adjusted analyses.

RESULTS

13a) CONSORT: For each group, the numbers of participants who were randomly assigned, received intended treatment, and were analysed for the primary outcome

Details of the numbers of participants who were randomly assigned, received intended treatment, and were analysed for the primary outcome are described in figure 3 (flow diagram) and table 2.

13b) CONSORT: For each group, losses and exclusions after randomisation, together with reasons

This is described in a consort flow diagram (figure 3).

13b-i) Attrition diagram

We have described this information in the text: "Out of the 48 intervention group participants 40% completed the intervention, 25% progressed up to stage 3 and 17 (35%) did not progress past stage 2. The mean (SD, range) number of logins to the programme was 18.68 (13.13, 1-51), an average of 3 logins per week per participant."

14a) CONSORT: Dates defining the periods of recruitment and follow-up

"Participant recruitment and outcome follow-ups were carried out from September 2008 to February 2010."

14a-i) Indicate if critical "secular events" fell into the study period

No critical 'secular events' occurred during the study period.

14b) CONSORT: Why the trial ended or was stopped (early)

The trial was not ended or was not stopped early.

15) CONSORT: A table showing baseline demographic and clinical characteristics for each group

Table 1 shows baseline demographic characteristics and table 2 shows baseline clinical characteristics.

15-i) Report demographics associated with digital divide issues

We have described age, and gender. However we did not collect data on education level, SES, or computer literacy level of the participants.

16a) CONSORT: For each group, number of participants (denominator) included in each analysis and whether the analysis was by original assigned groups

16-i) Report multiple "denominators" and provide definitions

Table 2 outlines the number of participants in each group included in the data analysis.

16-ii) Primary analysis should be intent-to-treat

"Data was analysed using intention to treat analysis; all participants with data available were included in data analysis according to the group first assigned at randomisation regardless of intervention compliance or adherence."

17a) CONSORT: For each primary and secondary outcome, results for each group, and the estimated effect size and its precision (such as 95% confidence interval)
For each primary and secondary outcome analysis the estimated effect size and its precision (such as 95% confidence interval) have been included, this is reported in both the text and in table 2.
17a-i) Presentation of process outcomes such as metrics of use and intensity of use
"Out of the 48 intervention group participants 40% completed the intervention, 25% progressed up to stage 3 and 17 (35%) did not progress past stage 2. The mean (SD, range) number of logins to the programme was 18.68 (13.13, 1-51), an average of 3 logins per week per participant."

However we did not collect data on 'average session length'.
17b) CONSORT: For binary outcomes, presentation of both absolute and relative effect sizes is recommended
This data was not collected.
18) CONSORT: Results of any other analyses performed, including subgroup analyses and adjusted analyses, distinguishing pre-specified from exploratory
Medium term effects have been reported: "Medium Term Intervention Effects
There were significantly lower levels of angina frequency (P=.026, 95% CI: 1.89:29.41, ES=0.63) and increased social QOL score (P=.018, 95% CI: 0.05:0.54, ES=0.60) favouring the intervention group at the 6 month follow up. In addition, the intervention group demonstrated increased emotional QOL score (P=.064, 95% CI: -0.02:0.62, ES=0.46) and decreased anxiety (P=.058, 95% CI: -2.60:0.04, ES=0.47) compared to the control group, these changes approached statistical significance. In contrast there were no significant medium term intervention effects in daily steps (P=.147, 95% CI:-358: 2324, ES=0.24), daily EE (P=.137,95% CI: -35.17:250.47, EE=0.38), DSA (P=.199, 95% CI: 0.190:0.205, ES=0.55), DMA (P=.244, 95% CI: 0.244: 0.261, ES=0.55), weight (P=.136, 95% CI: -2.46:0.34, ES=0.35), body fat % (P=1.00,95% CI: -3.81:3.81, ES=0.00), SBP (P=.531, 95% CI: -4.84:9.29, ES=0.15), DBP (P=.911,95% CI: -4.80:4.29, ES=0.03), fat intake (P=.279, 95% CI: -6.12:1.80, ES=0.30), fibre intake (P=.246, 95% CI: -2.23:8.53, ES=0.29), depression (P=.153, 95% CI: -2.11:0.34, ES=0.35), self-efficacy (P=.719, 95% CI: -2.32:3.34, ES=0.09), physical QOL score (P=.241, 95% CI: -0.11:0.43, ES=0.29), physical limitations (P=.710, 95% CI: -7.20:10.50, ES=0.08), angina stability (P=.582, 95% CI: -13.72:24.18, ES=0.13), treatment satisfaction (P=.724, 95% CI: -15.31:10.69, ES=0.08), or disease perception (P=.576, 95% CI: -8.41:14.99, ES=0.17). Even though there were no significant intervention effects present for many of the outcome measures it should be acknowledged that at the 6-month follow-up the intervention group showed trends of improved levels of baseline daily steps, EE, DSA, DMA, and weight while the control group declined at the 6-month follow-up."

18-i) Subgroup analysis of comparing only users
This type of analysis was not carried out.
19) CONSORT: All important harms or unintended effects in each group
"Unexpectedly there was also a significantly greater reduction in SBP in the control group compared to the web-based cardiac rehabilitation group (P=. 001, 95% CI: 2.99:13.91, ES=0.68)."
19-i) Include privacy breaches, technical problems
This did not occur in this study.
19-ii) Include qualitative feedback from participants or observations from staff/researchers
We are planning to publish our qualitative findings separately.

DISCUSSION

20) CONSORT: Trial limitations, addressing sources of potential bias, imprecision, multiplicity of analyses
20-i) Typical limitations in ehealth trials
"Strengths and Limitations: This study evaluated the effects of an internet delivered self managed cardiac rehabilitation programme in an angina population with objectively measured physical activity as the primary outcome; a group seldom included within rehabilitation research or rehabilitation services despite current guidelines [32]. The researcher who collected outcome measures also delivered the intervention, this allows for potential bias as participants with particularly high CHD risk could have unintentionally been encouraged more than other participants, which could have influenced the trial results. In future trials researchers taking outcome measures should be blinded. In addition it is necessary to consider 'measurement reactivity', where measurement results in changes in the people being measured [33]. Even though the study measured physical activity objectively there still remains the possibility that participants may have adjusted behaviour whilst the activity monitor was worn. The physical activity measurement period was 2 days; at the time 2 days was the recommended monitoring period [34]. For future studies we would propose to wear the monitor for a longer period, ideally 7 days. The study did not achieve the changes in physical activity that the power calculation was based upon, in hindsight this would appear to be an ambitious target, as the power calculation was based on an intervention that was much more intense than the one described here. The data shows that the intervention was effective in the short term, and the benefit was sustained in some outcomes at the 6 month follow up, in the absence of access to the site or any ongoing support. In the future we would wish to study the impact of continued access to the site for an extended period compared to best usual care. Due to the limitations in funding we were unable to collect any cost effectiveness data or health care utilisation, which would be desirable in future studies. Additionally, it would be valuable to assess if this intervention has an impact on smoking behaviour. The current intervention does comprise a smoking cessation component, although the affect of this component was not examined in the current study as only 2 (4%) and 6 (13%) participants in the intervention and control group respectively were smokers at baseline. Future research should examine the intervention's impact on smoking cessation. The sample recruited in this study was mainly of a White British origin. While this is not reflective of the general population, it is in line with the ethnicity of patients currently receiving traditional cardiac rehabilitation as reported in a national audit; challenges remain to find an acceptable intervention for ethnic minorities [5]. It would also be useful in future studies to compare the outcomes of an angina population using the web-based rehabilitation programme to an angina population receiving traditional rehabilitation."

"In terms of the technological advances in health care the programme could also be developed into an application for use on a Smartphone, and thereby enable the programme to be available via mobile phone technology. Research examining the value of mobile phone based interventions in increasing physical activity has been evaluated in a meta-analysis conducted by Fanning et al [35] and provides support for interventions using mobile technology to increase physical activity behaviour."
21) CONSORT: Generalisability (external validity, applicability) of the trial findings
21-i) Generalizability to other populations
Before we are able to generalise the findings we propose that a larger, pragmatic trial is required: "A large, pragmatic trial is required to examine the effectiveness and cost-effectiveness of this intervention when embedded into clinical practice."
21-ii) Discuss if there were elements in the RCT that would be different in a routine application setting
There were no elements in the RCT that would be different in a route setting.
22) CONSORT: Interpretation consistent with results, balancing benefits and harms, and considering other relevant evidence
22-i) Restate study questions and summarize the answers suggested by the data, starting with primary outcomes and process outcomes (use)

"Summary of findings: This study demonstrated daily physical activity improved as identified by step counts (our primary outcome). We also found significant improvements in a range of secondary outcome measures derived from the monitor, most importantly a reduction in sedentary time and an increase in the time spent being moderately active. This change in activity is an important outcome for this study as an important component of the site is to encourage daily exercise, most commonly walking. Even though the changes are not significantly better at 6 months there is a trend for the intervention group to remain improved compared to the control group, of which the effect sizes ranged from small to medium. This is in the absence of continued access to the site or any on-going support. At the 6-week follow-up we also observed important changes in weight, self-efficacy, emotional QOL score, and angina symptoms. We also observed significant changes at 6 months in angina symptoms and social QOL score."

22-ii) Highlight unanswered new questions, suggest future research

Unanswered, new questions and suggestions for future research have been described within the discussion's 'strengths and limitations' section: "Strengths and Limitations: This study evaluated the effects of an internet delivered self managed cardiac rehabilitation programme in an angina population with objectively measured physical activity as the primary outcome; a group seldom included within rehabilitation research or rehabilitation services despite current guidelines [32]. The researcher who collected outcome measures also delivered the intervention, this allows for potential bias as participants with particularly high CHD risk could have unintentionally been encouraged more than other participants, which could have influenced the trial results. In future trials researchers taking outcome measures should be blinded. In addition it is necessary to consider 'measurement reactivity', where measurement results in changes in the people being measured [33]. Even though the study measured physical activity objectively there still remains the possibility that participants may have adjusted behaviour whilst the activity monitor was worn. The physical activity measurement period was 2 days; at the time 2 days was the recommended monitoring period [34]. For future studies we would propose to wear the monitor for a longer period, ideally 7 days. The study did not achieve the changes in physical activity that the power calculation was based upon, in hindsight this would appear to be an ambitious target, as the power calculation was based on an intervention that was much more intense than the one described here. The data shows that the intervention was effective in the short term, and the benefit was sustained in some outcomes at the 6 month follow up, in the absence of access to the site or any ongoing support. In the future we would wish to study the impact of continued access to the site for an extended period compared to best usual care. Due to the limitations in funding we were unable to collect any cost effectiveness data or health care utilisation, which would be desirable in future studies. Additionally, it would be valuable to assess if this intervention has an impact on smoking behaviour. The current intervention does comprise a smoking cessation component, although the affect of this component was not examined in the current study as only 2 (4%) and 6 (13%) participants in the intervention and control group respectively were smokers at baseline. Future research should examine the intervention's impact on smoking cessation. The sample recruited in this study was mainly of a White British origin. While this is not reflective of the general population, it is in line with the ethnicity of patients currently receiving traditional cardiac rehabilitation as reported in a national audit; challenges remain to find an acceptable intervention for ethnic minorities [5]. It would also be useful in future studies to compare the outcomes of an angina population using the web-based rehabilitation programme to an angina population receiving traditional rehabilitation."

"In terms of the technological advances in health care the programme could also be developed into an application for use on a Smartphone, and thereby enable the programme to be available via mobile phone technology. Research examining the value of mobile phone based interventions in increasing physical activity has been evaluated in a meta-analysis conducted by Fanning et al [35] and provides support for interventions using mobile technology to increase physical activity behaviour."

"We would propose to offer access for a longer period of time. There may also be opportunities to explore the value of this intervention as an alternative to conventional rehabilitation for those individuals who have suffered an acute cardiac event, or indeed angina to increase the choice and scope of cardiac rehabilitation."

Other information

23) CONSORT: Registration number and name of trial registry

"Clinical Trial Registration: http://www.controlled-trials.com/ISRCTN90110503/oscar -ISRCTN90110503 [1]."

24) CONSORT: Where the full trial protocol can be accessed, if available

"Clinical Trial Registration: http://www.controlled-trials.com/ISRCTN90110503/oscar -ISRCTN90110503 [1]."

25) CONSORT: Sources of funding and other support (such as supply of drugs), role of funders

"The research was funded by the National Institute for Health Research (NIHR) Collaboration for Leadership in Applied Health Research and Care East Midlands (CLAHRC EM), and took place at the University Hospitals of Leicester NHS Trust. The views expressed are those of the authors and not necessarily those of the NHS, the NIHR or the Department of Health."

X26-i) Comment on ethics committee approval

"Ethics: The study protocol gained ethical approval was granted by the National Health Service Research Ethics Service (ref: 08/H1210/84) and by Coventry University. The trial was registered as The OSCAR Trial, ISRCTN90110503, http://www.controlled-trials.com/ISRCTN90110503/oscar [1]."

x26-ii) Outline informed consent procedures

"The initial home visit was arranged at a time most convenient for the participant. During the home visit the researcher (RD) explained that the purpose of the study was to investigate the effectiveness of a web-based intervention, described study details, took participant consent."

X26-iii) Safety and security procedures

We did not include any safety or security procedures.

X27-i) State the relation of the study team towards the system being evaluated

"Conflicts of Interest: The only study team member related to the intervention being evaluated is SS who participated in the design of the intervention evaluated in this manuscript. SS derives no financial benefit from the development of the intervention."
